# Posterior Reversible Encephalopathy Syndrome Caused by Renovascular Hypertension in a Solitary Functioning Kidney: Successful Treatment With Renal Artery Stenting

**DOI:** 10.7759/cureus.102381

**Published:** 2026-01-27

**Authors:** Hiromichi Ueno, Tetsu Miyamoto, Yuki Tsuda, Masaharu Kataoka

**Affiliations:** 1 Department of Physiology, University of Occupational and Environmental Health, Kitakyushu, JPN; 2 Second Department of Internal Medicine, University of Occupational and Environmental Health, Kitakyushu, JPN; 3 Department of Cardiology, Kitakyushu City Yahata Hospital, Kitakyushu, JPN

**Keywords:** posterior reversible encephalopathy, renal angioplasty, renal artery stenosis, renovascular hypertension, solitary functioning kidney

## Abstract

Posterior reversible encephalopathy syndrome (PRES) can develop in the setting of severe hypertension and may present with seizures and altered consciousness. We report the case of an 81-year-old woman who presented with impaired consciousness and generalized seizures during a hypertensive crisis. Brain magnetic resonance imaging revealed bilateral parieto-occipital subcortical hyperintensities. Diagnostic imaging revealed severe proximal stenosis of the right renal artery, complete occlusion of the left renal artery, and marked atrophy of the left kidney, suggesting renovascular hypertension due to renal artery stenosis in a solitary functioning kidney. Based on these findings, PRES secondary to renovascular hypertension was diagnosed, and percutaneous renal angioplasty was performed, resulting in stabilization of blood pressure and resolution of neurological symptoms. This case highlights the importance of considering renovascular hypertension as a reversible cause of PRES, especially in patients with a solitary kidney and marked blood pressure elevation and variability.

## Introduction

Posterior reversible encephalopathy syndrome (PRES) is a neurotoxic state characterized by vasogenic edema predominantly affecting the posterior cerebral white matter and is often reversible with timely treatment. However, delayed recognition may result in irreversible neurological damage such as infarction or hemorrhage [1]. PRES is most commonly associated with hypertensive emergencies, renal failure, autoimmune diseases, and exposure to immunosuppressive or cytotoxic agents, particularly calcineurin inhibitors [2-4]. The pathophysiology is believed to involve abrupt elevations and fluctuations in blood pressure exceeding the upper limits of cerebral autoregulation, resulting in endothelial dysfunction and vasogenic edema formation [3,5]. Therefore, timely identification and correction of the underlying cause are essential to prevent permanent neurologic sequelae [1,5].

Renal artery stenosis (RAS) is a well-recognized cause of secondary hypertension, particularly in elderly individuals with atherosclerosis, and accounts for approximately 1%-5% of hypertension cases [6]. Although randomized controlled trials such as Angioplasty and Stenting for Renal Artery Lesion (ASTRAL) and Cardiovascular Outcomes in Renal Atherosclerotic Lesions (CORAL) failed to demonstrate the superiority of percutaneous transluminal renal angioplasty (PTRA) over optimal medical therapy in unselected patients, more recent analyses suggest that specific high-risk individuals, such as those with flash pulmonary edema, rapid deterioration in renal function, or treatment-resistant hypertension, may benefit from revascularization [7,8]. Among these high-risk categories, renovascular disease affecting a solitary functioning kidney presents a particularly precarious situation with a high risk of hypertensive crises and progressive renal dysfunction due to lack of contralateral compensation.

Although rare, PRES secondary to renovascular hypertension has been reported, including in patients with bilateral RAS or RAS involving a solitary functioning kidney. Such cases highlight the importance of early recognition of cerebral complications and timely revascularization to reverse both neurological and renal dysfunction [9]. Here, we report a rare case of PRES caused by renovascular hypertension in a solitary functioning kidney, which was successfully treated with PTRA.

## Case presentation

In May 2015, an 81-year-old woman, previously treated elsewhere for hypertension accompanied by headache and nausea, was transferred to our hospital because of new-onset disturbed consciousness, generalized seizures, and severe hypertension (230/126 mmHg). On admission, her blood pressure was 175/98 mmHg, heart rate 96 beats/minute, and oxygen saturation 96% on room air. Her Glasgow Coma Scale score was E3V1M4, while no focal neurological deficits were observed. Cardiac auscultation revealed a grade II/VI systolic murmur at the right second intercostal space, and pulmonary examination was unremarkable. Laboratory tests showed chronic kidney disease stage 4, with a serum creatinine level of 1.72 mg/dL and an estimated glomerular filtration rate (eGFR) of 22.4 mL/minute/1.73 m².

**Figure 1:**
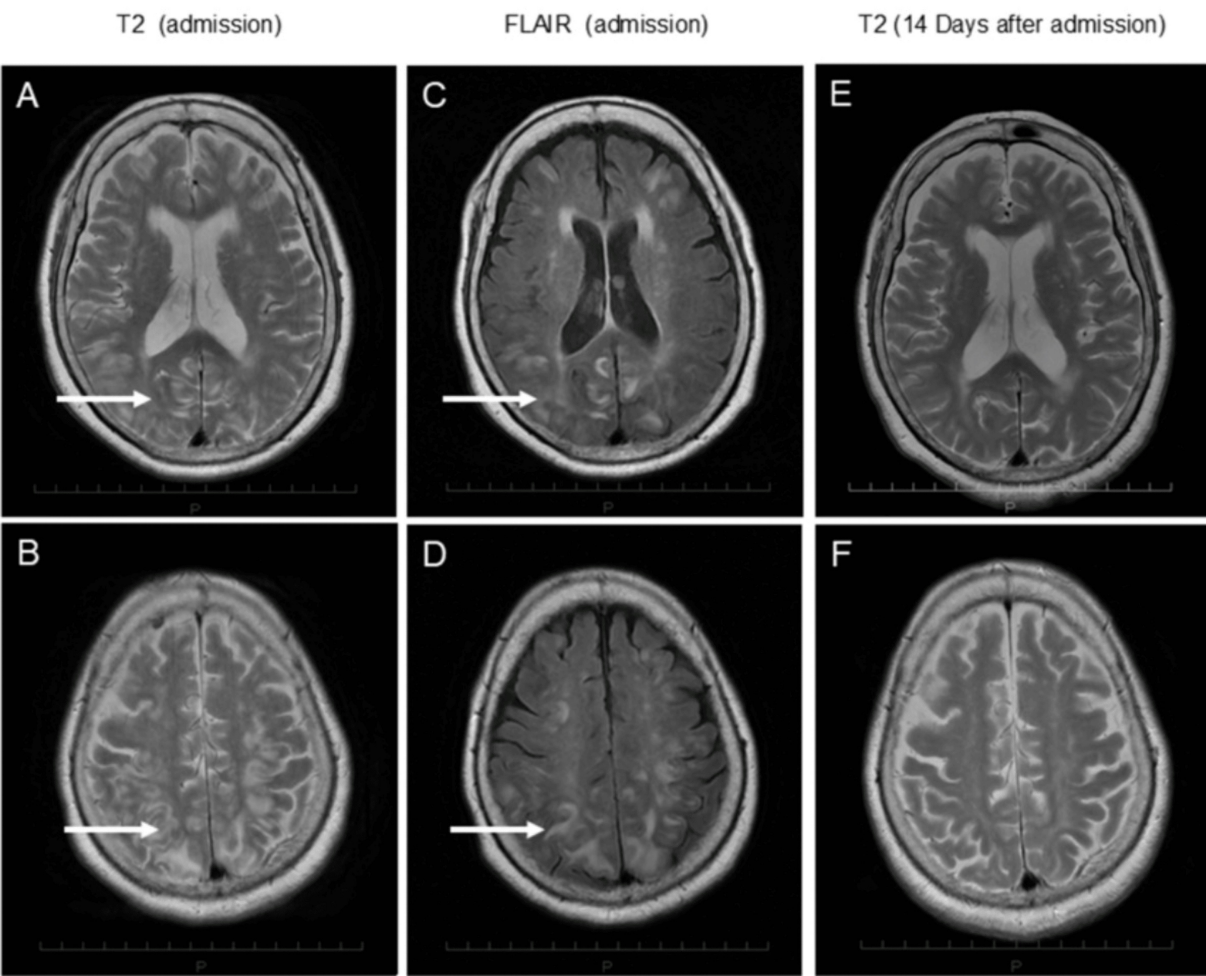
Brain MRI showing posterior vasogenic edema T2-weighted images (A,B) and FLAIR images (C,D) showing bilateral parieto-occipital subcortical hyperintensities consistent with vasogenic edema (white arrows). Follow-up MRI on day 14 demonstrating marked resolution of the lesions (E,F) MRI, magnetic resonance imaging; FLAIR, fluid-attenuated inversion recovery

Brain magnetic resonance imaging (MRI) demonstrated bilateral parieto-occipital subcortical hyperintensities consistent with PRES (Figures 1A-1D). After intravenous nicardipine administration and bed rest, her level of consciousness improved the following day. Long-term antihypertensive therapy was subsequently intensified, and four oral antihypertensive agents, azilsartan (40 mg/day), trichlormethiazide (1 mg/day), benidipine (4 mg/day), and nifedipine (80 mg/day), were administered in combination with dietary salt restriction, achieving partial blood pressure control. Follow-up MRI on day 14 showed marked improvement of the vasogenic edema (Figures 1E, 1F). Nevertheless, severe hypertension with marked blood pressure variability (systolic 130-200 mmHg; diastolic 50-100 mmHg) persisted.

Renal ultrasonography revealed complete occlusion and marked atrophy of the left kidney, while the right renal artery exhibited accelerated flow with turbulence (peak systolic velocity 2.83 m/s; renal-aortic ratio 4.6). Plasma renin activity was elevated (3.8 ng/mL/hour; reference range 0.8-2.0), whereas plasma aldosterone levels were suppressed (<10 pg/mL). Based on these findings, PRES secondary to renovascular hypertension caused by right RAS in a solitary functioning kidney was diagnosed, and the risk of PRES recurrence due to persistent blood pressure instability was considered high.

**Figure 2:**
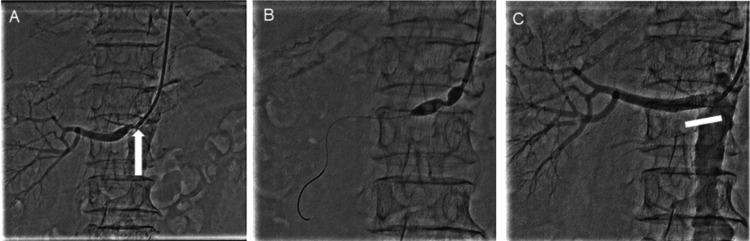
Right renal artery ostial stenosis and successful stent placement Right renal angiography showing severe ostial stenosis of the renal artery (A, arrow). Balloon angioplasty (B) followed by stent placement (C, rectangle) successfully restored renal arterial flow

**Figure 3:**
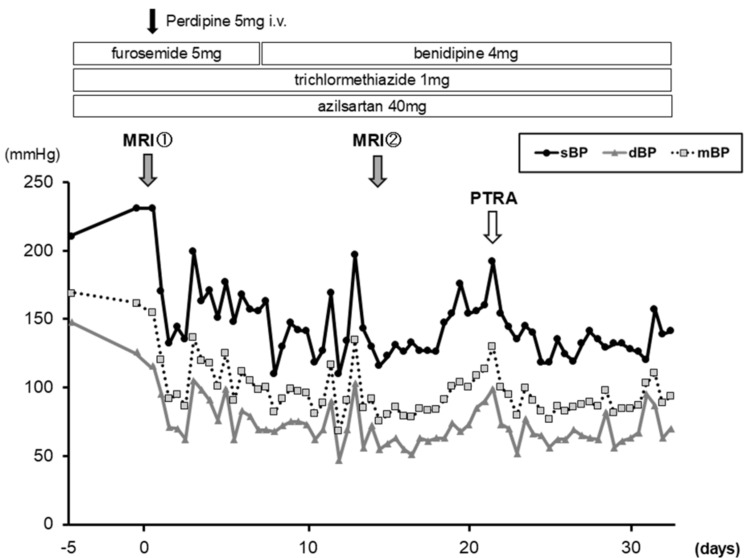
Blood pressure stabilization after renal artery angioplasty Time course of blood pressure before and after PTRA, demonstrating marked stabilization following the procedure. Initiation and intensification of antihypertensive therapy, as well as the timing of MRI examinations (on admission and on day 14), are indicated sBP, systolic blood pressure; mBP, mean blood pressure; dBP, diastolic blood pressure; PTRA, percutaneous transluminal renal angioplasty; MRI, magnetic resonance imaging

Given the refractory hypertension and clinical instability, endovascular treatment was deemed appropriate. PTRA was performed on hospital day 21. Angiography demonstrated 90% ostial stenosis of the right renal artery (Figure 2A). The lesion was dilated using a 7.0/20 mm SHIDEN® HP balloon (Kaneka, Tokyo, Japan), followed by deployment of a 6.0/18 mm Express™ Vascular SD stent (Boston Scientific, Marlborough, MA) (Figures 2B, 2C). After the procedure, blood pressure stabilized, headache resolved, and renal function (serum creatinine 1.87 mg/dL, eGFR 20.48 mL/minute/1.73 m²) was preserved (Figure 3). The patient was discharged on hospital day 33 with stable hemodynamics. At three-month follow-up after discharge, the patient remained neurologically asymptomatic, with stable blood pressure control and no further deterioration of renal function.

## Discussion

This case illustrates a rare presentation of PRES secondary to resistant renovascular hypertension caused by severe RAS in a solitary functioning kidney. PRES commonly presents with seizures, headache, visual disturbance, and altered mental status, and is frequently associated with hypertensive emergencies, renal dysfunction, or cytotoxic drug exposure [1,2]. The prevailing pathophysiological mechanism involves abrupt elevations and fluctuations in blood pressure exceeding the limits of cerebral autoregulation, leading to endothelial dysfunction and vasogenic edema, predominantly in the posterior cerebral circulation [4].

Renovascular hypertension accounts for approximately 1%-5% of secondary hypertension cases and is most often caused by atherosclerotic RAS. Although large randomized trials such as ASTRAL and CORAL did not demonstrate a clear overall benefit of revascularization over medical therapy, subsequent analyses and guideline statements emphasize that selected high-risk phenotypes may benefit from intervention [6,7]. Importantly, renal prognosis is substantially worse in renovascular disease involving a solitary functioning kidney compared with unilateral disease in patients with two kidneys, supporting early consideration of revascularization in unstable cases [8].

Although intervention in a solitary functioning kidney carries higher procedural stakes due to the absence of contralateral renal reserve, previous studies have shown that PTRA can be performed safely in experienced centers and may improve refractory hypertension while preserving renal function [10]. Consistent with this approach, the ACC/AHA/SCAI/SIR/SVM 2018 Appropriate Use Criteria for Peripheral Artery Intervention consider renal artery revascularization appropriate for patients with hemodynamically significant RAS accompanied by resistant hypertension or progressive renal dysfunction, including those with a solitary functioning kidney [5].

The coexistence of PRES and renovascular disease has been reported in both adults and children, suggesting that renovascular mechanisms can precipitate cerebral autoregulatory failure across age groups [10,11]. Our case adds to the existing literature by documenting a fully reversible episode of PRES associated with severe atherosclerotic RAS in a solitary functioning kidney. Clinicians should recognize that PRES may represent an early and reversible manifestation of renovascular hypertension, and timely endovascular intervention, when indicated, can lead to favorable neurological and renal outcomes. This case highlights the vulnerability of a solitary functioning kidney to marked blood pressure fluctuations and hypertensive crises. Although PRES associated with bilateral RAS has been previously reported, comparable cases involving a solitary functioning kidney remain extremely limited in the literature. While the underlying pathophysiological mechanism, namely, impaired blood pressure autoregulation leading to vasogenic edema, is likely similar to that observed in bilateral RAS, the present case underscores a distinct clinical context characterized by the absence of contralateral renal reserve. In this case, rapid revascularization with PTRA improved severe and labile blood pressure without deterioration of renal function, resulting in complete neurological recovery.

## Conclusions

This case highlights the importance of considering renovascular hypertension in patients with PRES secondary to severe, treatment-resistant hypertension. It also demonstrates that PTRA may be an effective treatment option for renovascular hypertension in patients with a solitary functioning kidney complicated by PRES. Early detection and consistent blood pressure control are essential in such cases to prevent irreversible neurological and renal damage.
